# The genome and transcriptome of the pine saprophyte *Ophiostoma piceae*, and a comparison with the bark beetle-associated pine pathogen *Grosmannia clavigera*

**DOI:** 10.1186/1471-2164-14-373

**Published:** 2013-06-02

**Authors:** Sajeet Haridas, Ye Wang, Lynette Lim, Sepideh Massoumi Alamouti, Shaun Jackman, Rod Docking, Gordon Robertson, Inanc Birol, Jörg Bohlmann, Colette Breuil

**Affiliations:** 1 Department of Wood Science, University of British Columbia, Vancouver, BC V6T1Z4, Canada; 2 Canada’s Michael Smith Genome Sciences Centre, Vancouver, BC V5Z 4E6, Canada; 3 Michael Smith Laboratories, University of British Columbia, Vancouver, BC V6T 1Z4, Canada

**Keywords:** *Ophiostoma piceae*, Genome, Transcriptome, Wood-staining fungus, Saprobe

## Abstract

**Background:**

*Ophiostoma piceae* is a wood-staining fungus that grows in the sapwood of conifer logs and lumber. We sequenced its genome and analyzed its transcriptomes under a range of growth conditions. A comparison with the genome and transcriptomes of the mountain pine beetle-associated pathogen *Grosmannia clavigera* highlights differences between a pathogen that colonizes and kills living pine trees and a saprophyte that colonizes wood and the inner bark of dead trees.

**Results:**

We assembled a 33 Mbp genome in 45 scaffolds, and predicted approximately 8,884 genes. The genome size and gene content were similar to those of other ascomycetes. Despite having similar ecological niches, *O. piceae* and *G. clavigera* showed no large-scale synteny. We identified *O. piceae* genes involved in the biosynthesis of melanin, which causes wood discoloration and reduces the commercial value of wood products. We also identified genes and pathways involved in growth on simple carbon sources and in sapwood, *O. piceae*’s natural substrate. Like the pathogen, the saprophyte is able to tolerate terpenes, which are a major class of pine tree defense compounds; unlike the pathogen, it cannot utilize monoterpenes as a carbon source.

**Conclusions:**

This work makes available the second annotated genome of a softwood ophiostomatoid fungus, and suggests that *O. piceae*’s tolerance to terpenes may be due in part to these chemicals being removed from the cells by an ABC transporter that is highly induced by terpenes. The data generated will provide the research community with resources for work on host-vector-fungus interactions for wood-inhabiting, beetle-associated saprophytes and pathogens.

## Background

**Figure 1:**
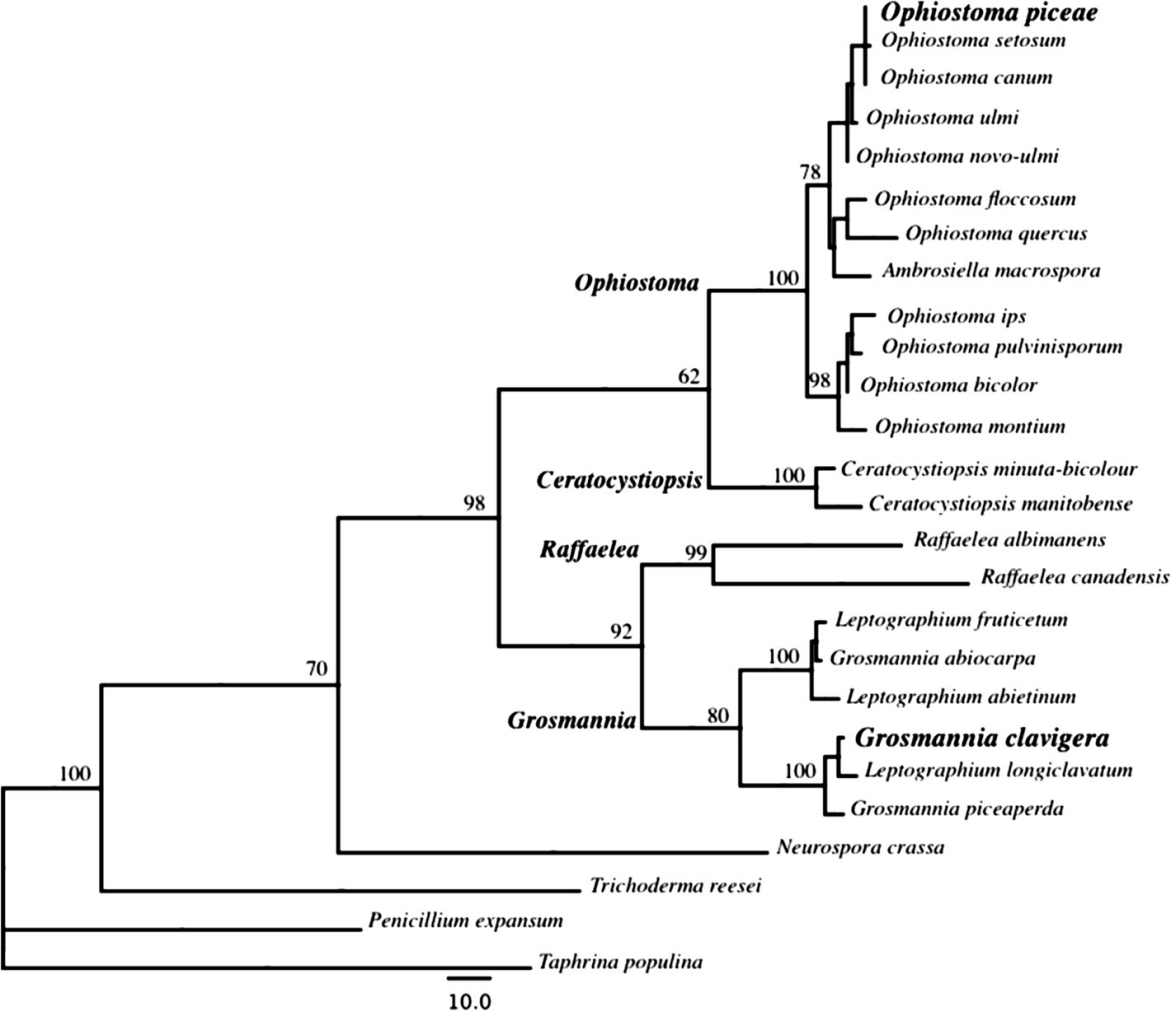
**ITSs-rDNA maximum parsimony tree places *O. piceae *within the Pezizomycotina. ***O. piceae* was originally described by Münch in 1907 [4]; this species and closely related species (e.g. *O. setosum*, *O. canum*, *O. novo-ulmi*, *O. ulmi* and *O. floccosum O. quercus*) are reported as the *O. piceae* complex. The numbers on the branches of the tree are bootstrap values based on 1000 replicates and the heuristic option [9].

**Figure 2:**
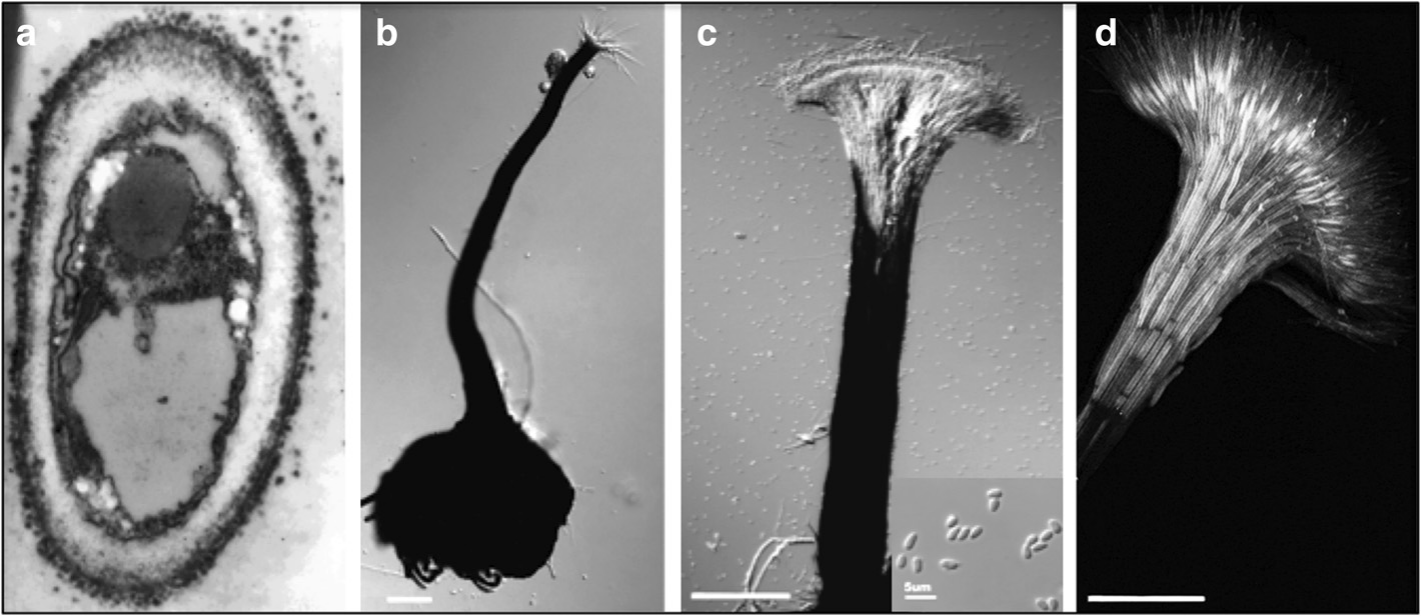
**Micrographs of *O. piceae *mycelium, sexual and asexual structures.** It grows as filamentous hyphae on solid media and as yeast like form in liquid media. *O. piceae* mycelium, perithecium and synemata are highly melanized while both asexual and sexual spores are not pigmented. The pigment accumulates as small black granules in the cell wall and the external sheath surrounding the hyphae (**a**: electron micrograph); Melanin is also present in fruiting body (perithecium) (**b**: light micrograph), as well as in the stem of the synemata (**c**: light micrograph; **d**: confocal micrograph), an aggregation of branched hyphae that produce abundant asexual spores. The fruiting bodies are easily obtained in artificial media or wood but require the pairing of two individuals with different mating types.

Pine trees and processed wood (lumber and logs) are colonized by ascomycete ophiostomatoid fungi that include pathogens and saprobes [1,2]. As they grow in the phloem and sapwood of the trees or in the sapwood of logs or lumber, most of these fungi produce a dark melanin pigment that causes a wood discoloration known as blue stain or sap stain. Ophiostomatoid sap stain fungi were first described more than 100 years ago [3,4] and have been recognized as an economic problem for forest industries worldwide. Currently, the group contains at least five genera that include *Ophiostoma* and *Grosmannia* (Figure 1). Ophiostomatoid fungi produce sticky sexual and asexual spores that are readily vectored by specific or generalist bark beetles that colonize trees or processed wood [5]. Before 1995, in Canada, *Ophiostoma* species were reported as the major cause of pine discoloration [1,6]. However, since 1995, in western Canada, the mountain pine beetle (MPB; *Dendroctonus ponderosae*) has expanded its range, and its fungal associates from the genera *Grosmannia* (mainly *G. clavigera* and *Leptographium longiclavatum*) and *Ophiostoma* (*O. montium*) have become the main cause of pine wood discoloration. As well, this beetle-fungal complex has killed large areas of pine trees in western North American conifer forests [7,8]. Wood discoloration is caused by melanin, a dark pigment that is synthesized inside the fungal cell and is released as small black globules into the cell wall and outside of the cell (Figure 2).

*O. piceae* is a saprobe that is dispersed by generalist bark beetles [10]. This fungal species has been reported in North America, Europe and Asia [5,6,11]. In contrast to *Grosmannia* species, which penetrate deeply into the sapwood of pine logs and reach the heartwood boundary, *O. piceae* is a more superficial sap stain fungus that becomes established in the outer two to three centimetres of sapwood [1,12,13]. Species in the *O. piceae* complex have retained the attention of wood industry researchers because they cause stain in processed wood and were the most commonly isolated species of sap stain fungi in Canadian saw mills [6]. In contrast to *G. clavigera*, which is specific to pine, *O. piceae* is able to grow not only on pine, but also on wood of other conifers in Canada, including black and white spruce, balsam fir and hemlock [6]. Because members of the *O. piceae* complex grow poorly on freshly cut pine logs and prefer the dryer environment of lumber or dead trees [1,13], their staining effects can be minimized by keeping logs frozen or saturated with water, or by prompt log processing. Green lumber can be protected by kiln drying to below 20% moisture content, or by chemical and biological treatments [14-16].

Both the pathogen *G. clavigera* and the saprophyte *O. piceae* acquire nutrients from pine species by secreting extracellular enzymes to break down large molecules like polysaccharides (e.g. hemicellulose and starch), proteins and lipids. They do not degrade wood and do not affect wood structural properties [1,16,17], so likely have limited or incomplete cellulolytic and/or lignolytic activities. However, in order to colonize conifers (e.g. lodgepole pine), fungi and their bark beetle vectors have to cope with the host’s preformed and induced defense chemicals, which include terpenoid and phenolic compounds [18-20]. Pathogens like *G. clavigera* have evolved mechanisms to overcome these defences [8,21]. However, the role of such host defence compounds in cut logs and lumber, where saprophytes like *O. piceae* are generally found, has not been reported. It is important to note that the composition of defence chemicals, especially terpenoids, varies with different pine genotypes across the landscape and can be affected by the environment [22]. Further, wood processing and drying affect the concentrations of chemicals in wood products, and so logs and lumber typically contain lower concentrations of the subset of terpenoids that are volatile [23,24].

In previous work we reported the annotated genome of the pine pathogen *G. clavigera*, and showed that this fungus is able to tolerate and utilize pine defence compounds, specifically terpenoids found in pine oleoresin [8,21]. Here, we report the annotated genome of the saprophyte *O. piceae*, and its gene expression responses in a range of growth conditions that include wood nutrients and host defence chemicals. We compare these results to corresponding results from *G. clavigera*, and highlight differences between a pathogen that colonizes and kills living pine trees and a saprophyte that lives on dead trees or processed wood. Neither fungus has a lignocellulolytic enzyme system that would allow it to degrade wood. Both fungi overcome terpenoid defence chemicals in their pine niches; however, only the pathogen, but not the saprophyte, can metabolize terpenoids as a carbon source. Both fungi have a similar ABC efflux transporter that is highly induced with monoterpene treatments. The functionality of the *O. piceae* transporter remains to be fully characterized.

## Results

### Genome assembly

We used ABySS [25] to assemble 100-nt reads from 200- and 700-nt insert Illumina HiSeq 2000 libraries, and ~300-nt reads from an 8-kb insert 454 Titanium (Table 1, Methods). The Illumina libraries provided >100× coverage for assembly and initial scaffolding, while the 454 reads supported long-range scaffolding. Refining this assembly with two iterations of Anchor resulted in a genome that consisted of 244 scaffolds, each of which was at least 1,000 nt in length. The assembly contained 335 false gaps represented by a single lowercase ‘n’ (see Methods). Of these, 219 were resolved by mapping Trinity-assembled RNA-seq transcripts to the genome using exonerate est2genome [26]. The remaining 116 gaps were resolved by using exonerate to find small overlaps (<5 bp) at the ends of contigs that were joined by an ‘n’.

**Table 1:** **Sequencing strategy for *O. piceae *genome**

**Sequencing technology**	**Read length (nt)**	**Insert length (nt)**	**Read pairs (Millions)**
Illumina Hiseq	100	200	87.8
Illumina Hiseq	100	700	32.2
454 Titanium	318 (median)	8000	0.3

We removed from the final assembly 187 scaffolds and contigs smaller than 10,000 bp (including gaps) that represented 1% of the assembly because they contained no genes or t-RNAs. The corrected 32.8-Mbp genome assembly consisted of 45 scaffolds. One percent of the genome consisted of 342 gaps (N’s). Half of the genome was in nine scaffolds that had an N50 of approximately 1.45 Mbp, while 90% was represented in 27 scaffolds that had an N90 of approximately 0.38 Mbp. Using CEGMA [27], we identified complete copies of 233 of 248 conserved eukaryotic genes and partial copies of an additional five, which suggests that our assembly represents 94% - 96% of the *O. piceae* gene space [28]. The genome characteristics of *O. piceae*, and three other ascomycetes [8,29,30] also in the class of the Sordariomycetes and found on dead tree and wood products are summarized in Table 2.

**Table 2:** **Characteristics of the *O. piceae *(Op) genome assembly and annotation and a comparison with other related genomes**

	**Op**	**Gc**^***a***^	**Nc (10)**^***b***^	**Tr**^***c***^
Genome size (Mbp)	32.8	30	41	33.5
Number of scaffolds	45	289	7 ^d^	87
N50 (Mbp)	1.45	2	1.56	1.12
Number of ungapped contigs	388	478	956	231
Genome GC content (%)	52.8	53.4	48.25	52.7
Non-coding genome (%)	54	54.28	56	
Number of genes	8,884	8,312	9,733	9,129
Median CDS length (bp)	1,401	1,350	1,673	1,299
Exon GC content (%)	59.7	60.4		57.8

a. *G. clavigera* (Gc); b. *Neurospora crassa* Sequencing Project, Broad Institute of Harvard and MIT (NC10). c*. Trichoderma reesei* (Tr). d. Chromosome numbers.

### Genome features and annotation

We used the Maker annotation pipeline [31] to predict genes. Within the annotated genome of *O. piceae*, we identified genes and gene families for secondary metabolite processing, cytochrome P450 as well as ABC transporters. We also identified homologous *O. piceae* and *G. clavigera* proteins based on reciprocal best BLAST hits. We further characterized the MAT idiomorph that is responsible for the mating type of the sequenced strain.

Maker predicted 8,884 proteins within our acceptance criteria (see Methods), of which 8,723 were at least 100 amino acids long. Almost 65% (5,786) of the predicted proteins encoded by the gene models had a known Pfam domain. Some of the major gene families in *O. piceae* are shown in Table 3. About a third of the predicted genes (3,026) had only one exon and only 1,283 transcripts were encoded by four or more exons. In this compact genome, genes, not including their upstream and downstream untranslated regions (UTRs) represent 45% of the assembly. Almost a quarter (1,984) of the predicted gene coding sequences (CDS) was within 500 bp of their respective neighbouring CDS, and almost half (4349) were within 1,000 bp of its neighbour. Our analysis predicted that 778 CDSs encode secreted proteins.

**Table 3:** **Major gene families in *O. piceae *(Op) and in three other ascomycetes**

**Gene family**	**Op**	Gc*	Nc*	Tr*
MFS transporters	289	227	161	236
ABC transporters	34	40	36	48
ATPases	308	349	356	352
NAD binding proteins	258	254	211	301
FAD binding proteins	130	146	122	144
Cytochrome P450s	45	54	43	73
Methyltransferases	112	159	126	125
Transcription factors	115	133	106	218
Glycoside hydrolases	140	126	168	170
Glycosyl transferases	63	64	76	79

*G. clavigera (Gc); *Neurospora crassa* (Nc) and *Trichoderma reesei* (Tr).

Although *O. piceae* and *G. clavigera* share hosts, cause sap-stain in pine, and are in sister clades in the Ophiostomatales [32] (Figure 1), their genomes showed no large-scale synteny (Additional file 1). This is consistent with synteny being lost with evolutionary time between members of the class Dothideomycetes [33]. Dothideomycetes is a sister clade of the class Sordariomycetes, which include *O. piceae* and *G. clavigera*, and we anticipate that similar synteny losses have occurred within this class. A BLAST comparison of the two predicted proteomes showed that 5,450 proteins were reciprocal best hits. These included most of the major metabolic functions. The *O. piceae* proteins with no significant homolog in the *G. clavigera* genome were overrepresented by protein kinases (GO:0004672), sequence-specific DNA binding RNA polymerase II transcription factors (GO: 0000981) and zinc ion binding proteins (GO:0008270) (Additional file 2). In addition, proteins involved in transmembrane transport (GO:0055085) were also significantly overrepresented in this group of 3,469 proteins (Additional file 2). Over 40% (1,397) of the *O. piceae* proteins with no evident homologs in *G. clavigera* were proteins of unknown function (predicted or hypothetical proteins). None of the six carboxylic ester hydrolases (GO:0052689) in the *O. piceae* genome had a homolog in the *G. clavigera* genome.

We searched for genes that may be involved in producing secondary metabolites (SMs). Such genes are typically organized as contiguous genomic clusters and can be identified by tools like SMURF [34], which uses hidden Markov models that consider genomic context and domain content. The first step in fungal SM biosynthesis is usually catalyzed by ‘backbone’ genes like nonribosomal peptide synthases (NRPSs), polyketide synthases (PKSs), hybrid NRPS-PKS enzymes, prenyltransferases, and terpene cyclases [34]. SMURF, which does not identify clusters containing terpene cyclases, identified thirteen backbone genes of which eleven are in SM clusters in *O. piceae* (Additional file 3), and nineteen genes in fourteen clusters in *G. clavigera*[35]. All the *O. piceae* SM genes have homologs in *G. clavigera*.

Melanin is a secondary metabolite that is produced by *O. piceae* and related species, but, as in *O. piceae*, the genes responsible for its production do not always occur in a cluster. Melanin is synthesized through the 1,8–dihydroxynaphthalene (DHN) pathway [36]. In *O. piceae* we identified a number of genes that were similar to genes that have major roles in the DHN pathway in *Ophiostoma*, *Grosmannia* and *Ceratocystis* species [12,37,38]. These genes included a PKS (OPP_00823), two reductases (OPP_02710, OPP_00820) and a scytalone dehydratase (OPP_07153). PKS catalyze both the elongation of five ketide subunits and the cyclization of these units to form the base ring of naphthalene. The first reductase (OPP_02710) converts 1,3,6,8-hydroxynaphthalene to scytalone, while the second (OPP_00820) transforms scytalone to vermelone.

*O. piceae* is a heterothallic species, so requires two individuals with different mating types for sexual reproduction and production of fertile fruiting bodies. Our genome annotation identified *O. piceae*’s MAT1-2 idiomorph (OPP_06680). A truncated MAT1-1 gene was next to the MAT1-2 gene, as in *Grosmannia* and related species [8,39,40]. We have produced perithecia for *O. piceae* by mating UAMH-11346 with UAMH- 11672 (Figure 2), and we have successfully amplified and sequenced the full length of the MAT1-1 loci in this latter strain; the alpha box (~978 bp) was missing in the sequenced strain.

### Gene expression patterns

To identify genes that may be critical for the saprophyte *O. piceae* to grow in the presence of the nutrients and defence chemicals that are characteristic of its natural pine sapwood substrate, we profiled gene expression for the fungus growing on solid agar media supplemented with simple carbon sources (i.e. sugars and lipids), with pine sawdust, or with pine terpenes (see Methods). Mapping the RNA-seq reads to the predicted gene models identified 7,157 genes that had an abundance of at least 10 FPKM (fragments per kilobase of exon per million fragments mapped) in any of the conditions tested.

**Figure 3:**
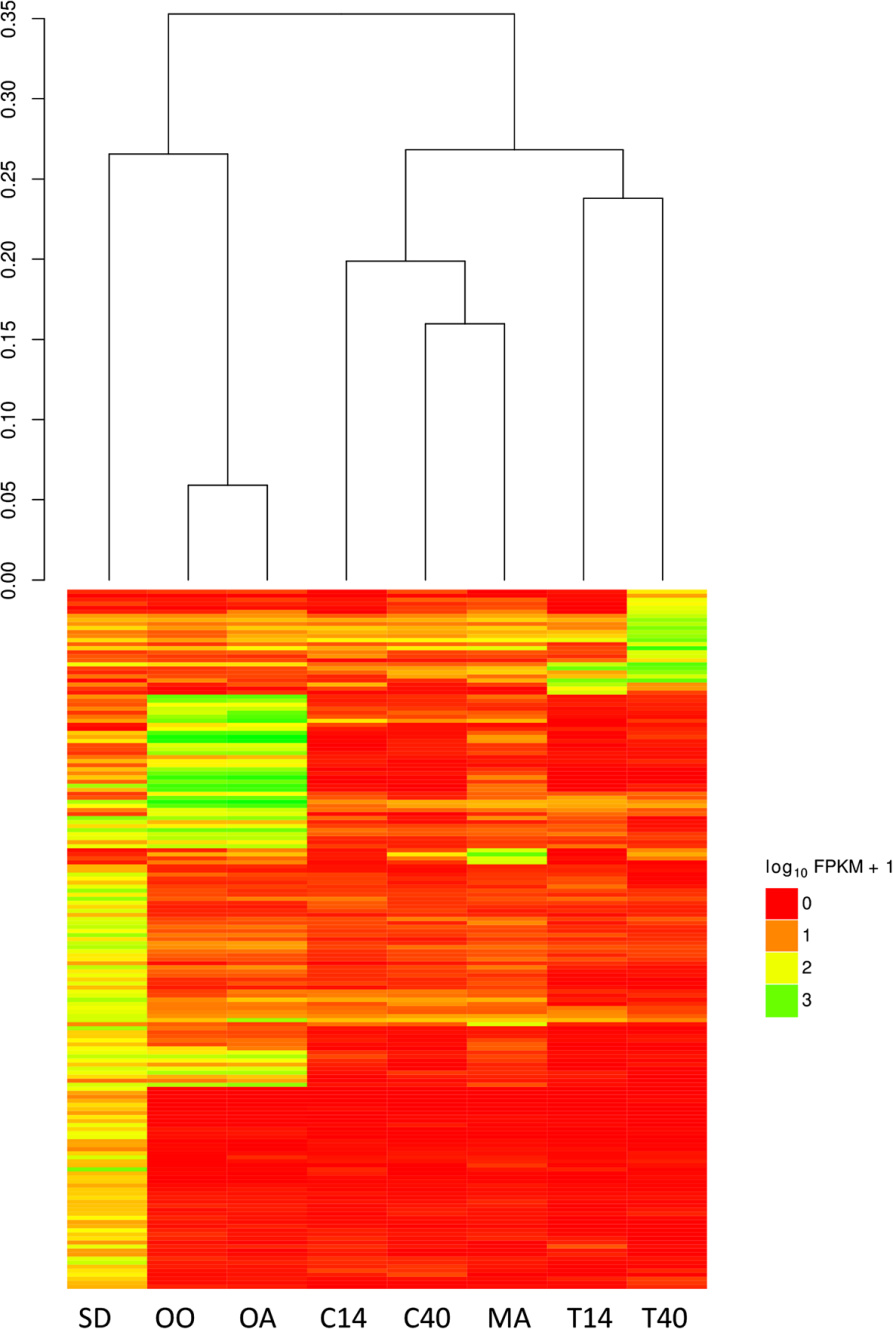
**Expression pattern of 173 selected genes of *O. piceae *and a dendrogram based on Jensen-Shannon distances between the conditions.** The expression of each gene (row) across the various growth conditions (column) is presented as log transformed values of fragments per kilobase of exon per million fragments mapped (FPKM). Red shows low expression and green shows high expression. Sawdust (SD); Triglyceride (TG/Olive oil, OO); Oleic acid (OA); CM at 14 hr (C14); CM at 40 hr (C40); Mannose (MA); CM + Terpene at 14 hr (T14); CM + Terpene at 40 hr (T40).

To select genes that were highly differentially regulated under different growth conditions, we required a gene to have an FPKM abundance that was at least ten times higher in a specific condition, or a related set of conditions, than in all other growth conditions. This approach identified 677 genes whose transcripts were differentially abundant in at least one growth condition (Additional file 4), and 173 genes whose transcripts were differentially abundant in only one condition (Figure 3). By manually comparing the set of 173 genes to functional information in the Gene Ontology (http://www.geneontology.org/) and to reference metabolic pathways KEGG (http://www.genome.jp), we identified pathways that were likely involved in the response of *O. piceae* to the growth conditions tested in our experiments. We added support for these pathways by manually identifying genes from the 677-gene set (Additional file 4) whose transcripts, while up-regulated, did not pass the stringent 10-fold filter used to identify the set of 173 genes.

We also assessed alternative transcript splicing across the range of growth conditions used for this study (Additional file 5). Splicing appeared not to be an important factor under these conditions.

### Growth on mannose, fatty acid (oleic acid) and triglycerides

Mannose is a simple monomeric epimer of glucose and can be readily utilized as a carbon source by *O. piceae*. We found five genes whose expression was at least ten times higher with mannose than in any other conditions tested (Additional file 4 shown light blue). These included two transporters, one oxidoreductase and two hypothetical proteins. Our data suggests that mannose uptake involves two transporters (OPP_03031, OPP_05665), and a simple isomerisation/ epimerization reaction by an oxidoreductase (OPP_00733) converts it into glucose. The function of two remaining up-regulated genes (OPP_02416, OPP_07274) is unknown.

We grew *O. piceae* on triglycerides and fatty acids, which are important lipid compounds in lodgepole pine sapwood, and are a major source of carbon for *O. piceae*[41]. Because most sources of triglycerides contain a small proportion of fatty acids, it was not surprising that most of the 129 genes whose transcripts were differentially abundant between these conditions were highly up-regulated in both of the conditions. Of the 25 up-regulated genes that were significantly induced only in these two conditions (shown in gold in Additional file 4), 18 were predicted to produce secreted proteins. Unexpectedly, the differentially up-regulated genes included no fungal lipases, which are necessary for the hydrolysis of triglycerides (Additional file 4). Twenty-three of the 25 up-regulated genes were predicted to be involved in the breakdown of carbohydrates and sugars; these included eight genes coding for secreted proteins in the glycoside hydrolase family and four genes for secreted proteins involved in carbohydrate and starch binding. We identified a transcription factor (OPP_02429) that showed significant up-regulation in the presence of triglycerides and oleic acid.

One of the genes differentially expressed between olive oil and oleic acid was a cytochrome P450 (OPP_02426) with a significantly higher expression with triglyceride than with fatty acid. Like its *G. clavigera* homolog (CMQ_5365; CYP630B18) and homologs in several other species including *Fusarium graminearum*, *Aspergillus niger*, *A. fumigates* and others, this gene is in close proximity to genes encoding a myo-inositol transporter, ARCA-like protein and a cytochrome P450 reductase [35].

### Growth on pine sapwood, a natural substrate for *O. piceae*

**Figure 4:**
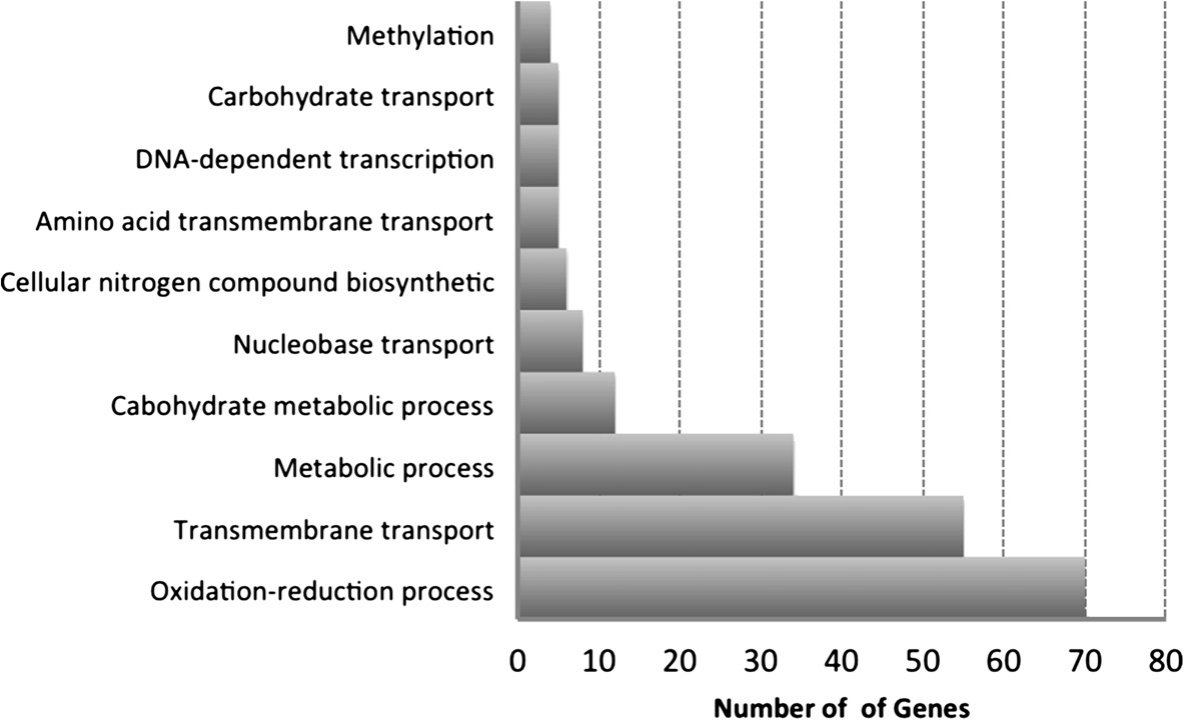
**Functional classification of up-regulated genes of *O. piceae *grown on sawdust using Blast2go.** Potential biological roles of transcribed proteins during growth experiments on different media were inferred by first identifying genes that were exclusively up-regulated (e.g. in sawdust), then associating the encoded protein sequences with biological processes using Blast2go.

*O. piceae* has slower growth rates than *G. clavigera* on rich media and on wood; for example, here, on MEA, *O. piceae* grows 5.8 mm/day, while in Wang et al. [21], *G. clavigera* grew 14 mm/day. Of the treatments used in this study, sawdust obtained by grinding pine sapwood was the closest to the natural substrate. It contains a variety of carbon sources including mannose, triglycerides and fatty acids. In this growth condition, 366 genes were up-regulated, 91 of which were up-regulated only in the presence of sawdust (Additional file 4). The subset of 91 genes was overrepresented in GO terms for transport (GO:0005215, GO:0006810; p < 0.0001) (Figure 4), which may reflect the complexity of the nutrient sources used by *O. piceae*. The up-regulated transporters included several allantoate, urea, hexose, iron and sugar transporters, and other major facilitator superfamily (MFS) transporters. As well, oxidoreductase genes that code for putative proteins involved in the modification of aromatic compounds, including phenolics, were highly up-regulated (e.g. P450s, dehydrogenases).

Among the 91 genes that were up-regulated on sawdust, 32 were found in 8 genomic clusters, each of which contained four to seven genes that may be co-regulated (Table 4). Three of the clusters contain a fungal-specific transcription factor, Zn2cys, which may be involved in primary and secondary metabolism and drug resistance [42]. Four of the clusters contain at least one of the following: a putative secreted salicylate dehydroxylase, an NAD-dependant epimerase, an alpha-mannosyltransferanse and an FAD–binding protein. An additional 22 genes that were up-regulated with sawdust were also up-regulated with triglycerides and oleic acid (dark blue in Additional file 4). The overall set of 113 genes (i.e. the 91 and the 22) was overrepresented in GO terms for secreted proteins (GO:0005576; p < 0.001) and carbohydrate metabolism (GO:0005975, GO:0030246; p < 0.001).

**Table 4:** Gene clusters up-regulated in sawdust

**Gene IDs**	**Putative function**	**Secreted**	**Log**_**2**_(FC)*
**Cluster 1**			
OPP_08738	Inositol monophosphatase	No	3.98
OPP_08737	Catabolic 3-dehydroquinase	No	5.78
OPP_08736	3-dehydroshikimate dehydratase	No	7.20
OPP_08735	Quinate dehydrogenase	No	5.65
**Cluster 2**			
OPP_06948	Allantoate permease	No	8.05
OPP_06946	Sarcosine oxidase	No	5.97
OPP_06944	Fungal-specific transcription factor domain protein	No	4.50
OPP_06943	Oxoglutarate 3-dioxygenase	No	8.75
**Cluster 3**			
OPP_07708	Sugar transporter	No	6.06
OPP_07707	Salicylate hydroxylase (salicylate 1-monooxygenase)	Yes	7.75
OPP_07706	NAD dependent epimerase	Yes	3.62
OPP_07705	Arylacetamide deacetylase	No	4.69
**Cluster 4**			
OPP_08830	Amidohydrolase family protein	No	8.98
OPP_08829	Aldehyde dehydrogenase	No	7.04
OPP_08827	FAD binding domain protein	Yes	6.25
OPP_08826	Retinol dehydrogenase 13	No	7.78
OPP_08825	Cytochrome p450	No	9.25
OPP_08824	General alpha-glucoside permease	No	7.98
**Cluster 5**			
OPP_07998	Xaa-pro dipeptidase	No	3.34
OPP_07997	Major facilitator superfamily transporter	No	4.21
OPP_07996	Hexose transporter	No	5.56
OPP_07995	Thymine dioxygenase	No	4.95
**Cluster 6**			
OPP_01495	N-carbamoyl-l-amino acid hydrolase	No	9.85
OPP_01494	Gal4-like transcription factor	No	6.74
OPP_01493	Class ii aldolase adducin domain-containing protein	No	9.97
OPP_01491	Isoflavone reductase family protein	Yes	6.49
**Cluster 7**			
OPP_05544	Hypothetical protein	No	10.82
OPP_05543	Alpha-mannosyltransferase	Yes	8.083
OPP_05542	Ethanolamine utilization protein	No	7.16
OPP_05541	C6 zinc finger domain containing protein	No	4.99
OPP_05540	Alpha-mannosyltransferase	No	8.78
**Cluster 8**			
OPP_02428	Myo-inositol transporter	No	7.42
OPP_02427	Arca-like protein	No	3.81
OPP_02426	Benzoate 4-monooxygenase cytochrome p450	No	4.45
OPP_02425	NADPH-cytochrome p450 reductase	No	5.12
OPP_02424	NAD binding rossmann fold	No	4.02

* FC = fold change.

One of the above eight up-regulated genomic clusters contained seven genes that were involved in metabolizing quinic acid (OPP_8732 to OPP_8738). These include a quinate permease, two regulatory genes (an activator and a repressor), and the four genes of the quinate/shikamate catabolic pathway [43,44]. The latter four catabolic genes (OPP_08735 to OPP_08738) suggest that *O. piceae* uses quinic acid in wood as a carbon source. While this gene cluster has been reported in many fungi, we were unable to find the cluster in *G. clavigera*. To confirm that *O. piceae* can use quinic acid, while *G. clavigera* cannot, we showed that the former, but not the latter, grows on YNB media with quinic acid as the sole carbon source (Additional file 6). Finally, we identified a secreted lipase (OPP_00605) with predicted triglyceride degradation activity, whose transcript abundance was at least 50-fold higher in the mycelium grown in pine sapwood than in the control mannose.

### Tolerance of pine tree defence chemicals

**Figure 5:**
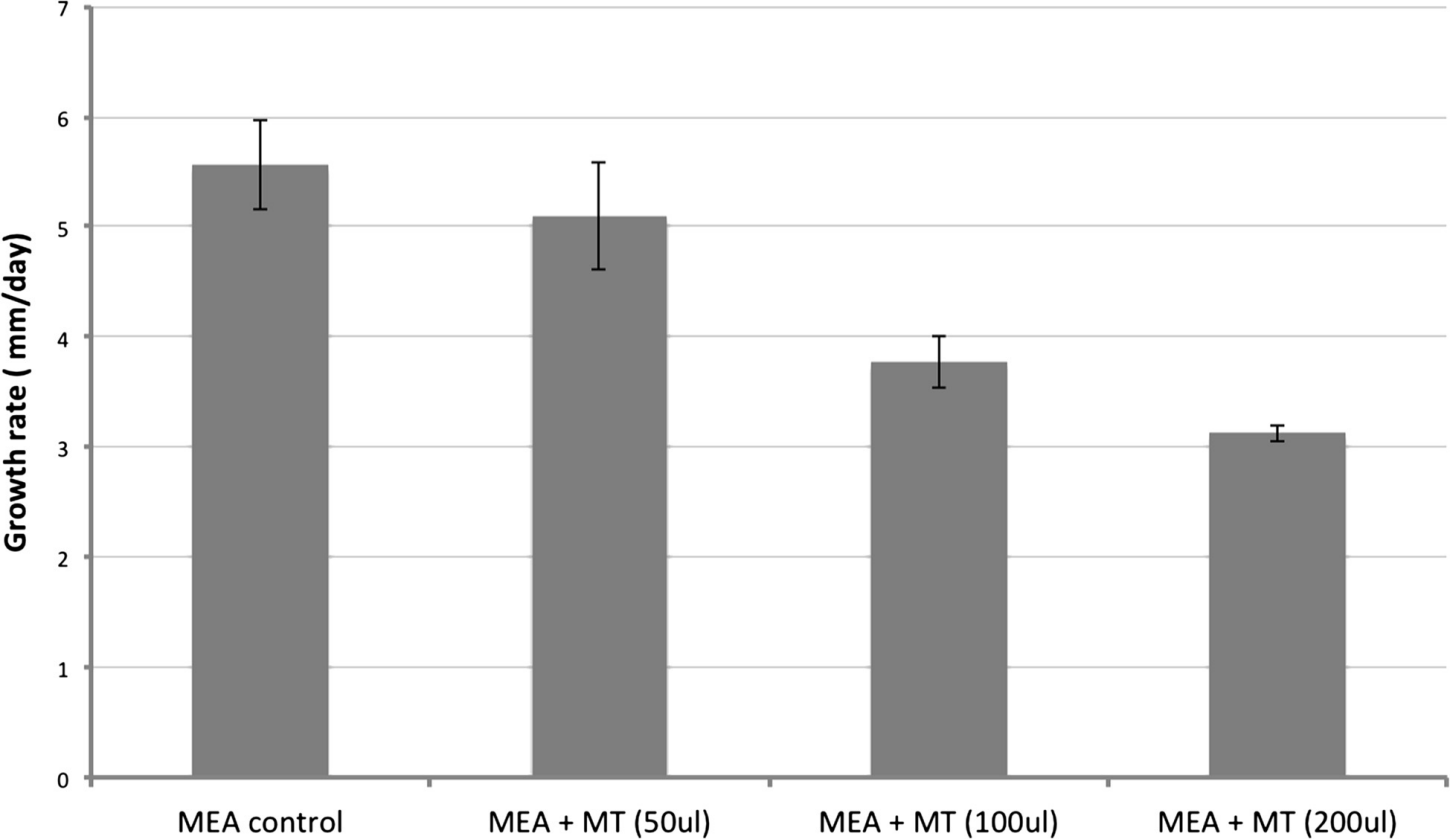
**Growth of *O. piceae *on malt extract agar (MEA) treated with various volumes of mixed monoterpenes (MT).** Fresh fungal mycelia were used as starting material and treated with 50, 100, 200 μl MT (equivalent to 0.35, 0.7, 1.4 g/l respectively). Colony diameters were measured daily. The growth rates were calculated as mm/day at linear stage. Results are average of 3 replicates; error bars are standard deviations. MT: (+)-limonene, (+)-3-carene, racemic α-pinene and (−)-β-pinene at a ratio of 5:3:1:1. Student t-test indicated that MT applied at 100 and 200 μl/plate inhibited fungal growth significantly, but not at 50 μl/plate ( P value cutoff 0.05).

*O. piceae* did not grow when a mixture of monoterpenes (MT) (i.e. (+)-limonene, (+)-3-carene, racemic a-pinene and (−)-ß-pinene at a ratio of 5:3:1:1) was the only carbon source in YNB. However, after a month of incubation in the presence of MT, the inocula resumed normal growth when they were transferred from YNB + MT to MEA. This suggests that *O. piceae* is able to survive in the presence of very high levels of monoterpenes. When the fungus was inoculated on MEA and treated with different amounts of MT, the growth rate was only significantly affected when at least 100 μl /plate (~ 0.7 g/l) of MT was added (Figure 5). For all MT treatments, the mycelia were more aerial and fluffy, while the asexual reproduction structures (i.e. formation of synemata) were highly inhibited (Additional file 7).

In order to identify genes involved in terpene tolerance, we grew *O. piceae* on CM and treated it with a mixture of terpenes as previously described in our studies with *G. clavigera*[8,21]. While the experiments for the two species were done at different times, we used the same conditions for both growth experiments and the same protocols for RNA extractions. We compared gene expression profiles of *O. piceae* after 14 h and 40 h treatments to profiles for untreated CM plates at the same time points. At 14 h, 295 genes were differentially abundant, 261 of which were down-regulated. While carbohydrate metabolism (GO:0005975) was associated with the down-regulated genes (p < 0.001), we were unable to identify any GO terms that were associated with the 34 up-regulated genes. After 40 h in the presence of terpenes, 264 genes were differentially abundant, 126 of which were up-regulated. While carbohydrate metabolism was still associated with down-regulated genes (p < 0.001), several transporters (OPP_06758, OPP_05515, OPP_03974, OPP_02103) were significantly up-regulated. In *G. clavigera*, which is able to utilize terpenes as a carbon source, more than 250 genes were up-regulated by at least 2-fold at 12 h and 36 h in the presence of terpenes [8]. Of the 34 *O. piceae* genes that were up-regulated at 14 h, 26 had homologs in *G. clavigera*, and nine of these *G. clavigera* genes were up-regulated at 12 h. Similarly, of the 126 *O. piceae* genes that were up-regulated at 40 h, 75 had *G. clavigera* homologs, twenty of which were up-regulated at 36 h.

**Figure 6:**
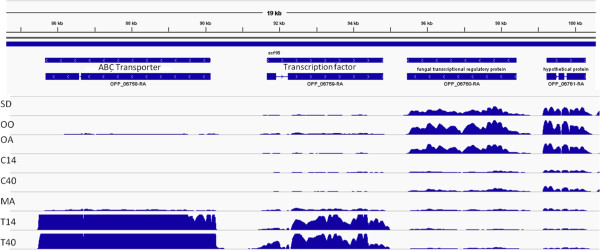
**Expression pattern of the most highly up-regulated ABC-G transporter (OPP_06758) and of a transcription factor (OPP_06759).** Relative transcript abundance of the gene OPP_06758 encoding an ABC transporter at 40 hr after terpene treatment. Sawdust (SD); Triglyceride (TG/Olive oil, OO); Oleic acid (OA); CM at 14 hr (C14); CM at 40 hr (C40); Mannose (MA); CM + Terpene at 14 hr (T14); CM + Terpene at 40 hr (T40).

**Figure 7:**
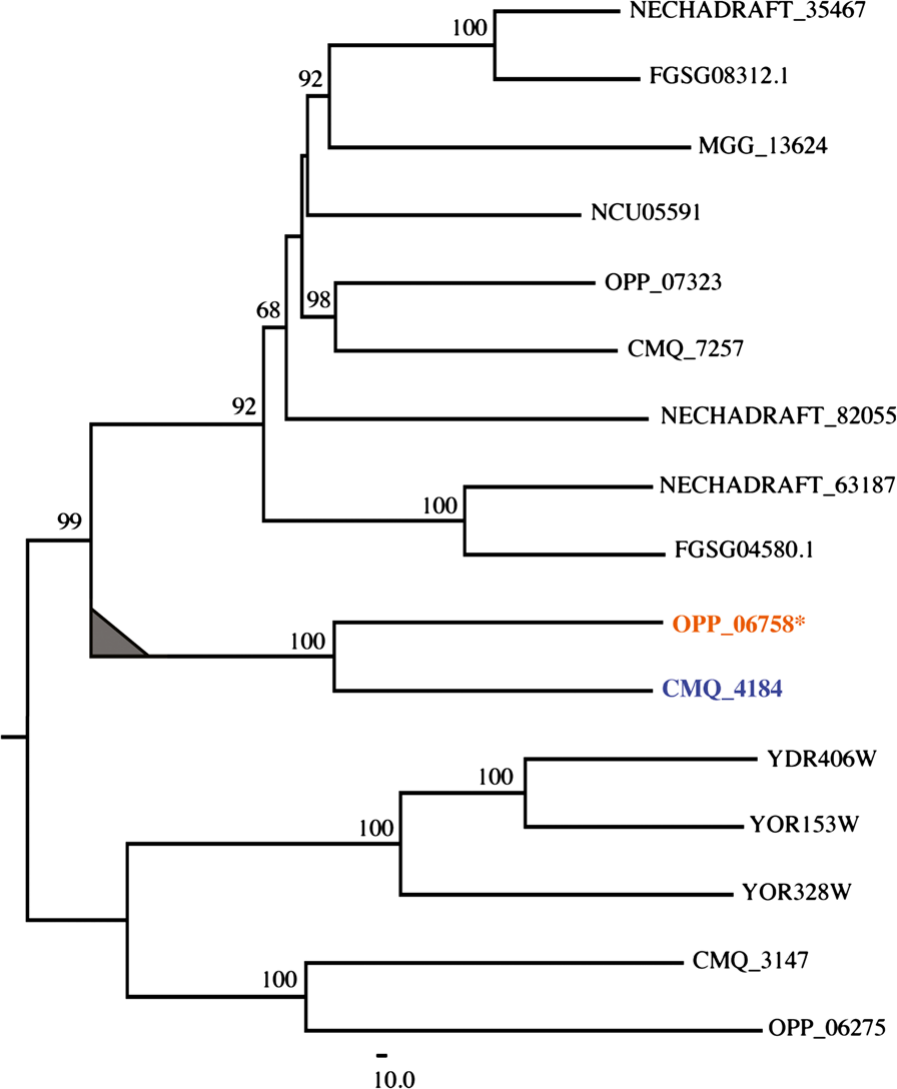
**Phylogenetic tree of ABC-G group I transporters in *O. piceae *(OPP_06758, orange) and *G. clavigera *(CMQ_4184, blue).** We included two other ABC transporters from *O. piceae* (OPP_06275, OPP_07323 ) and from *G*. *clavigera* (CMQ_3147 and CMQ_7257) and a subset of ABC transporters from ascomycete species**.** These species are: *Saccharomyces cerevisiae* (YOR328W, YOR153W, YDR406W); Pyrenomycetes like *Gibberella zea* (FGSG04580, FGSG08312), *Nectria Haematococca* (NECHADRAFT_63187, NECHADRAFT_35467, NECHADRAFT_82055), *Neurospora crassa* (NCU05591), *Magnaporthe grisea* (MGG_13624).

We found 26 *O. piceae* genes that were up-regulated only in the presence of terpenes, at one or both time points. Eighteen of these had *G. clavigera* homologs. The most highly up-regulated *O. piceae* gene (OPP_06758) encoded an ABC transporter that was homologous to the *G. clavigera* transporter (CMQ_4184; GcABC-G1) that confers terpene tolerance to the pathogen [21] (Figure 6). Approximately 1,500 bp upstream of the *O. piceae* ABC transporter is a gene (OPP_06759) encoding a transcription factor whose expression, like that of the transporter, was up-regulated only in the presence of terpenes (Figure 6). We found that the *O. piceae* OPP_06758 and the *G. clavigera* CMQ_4184 (GcABC-G1) ABC transporters were placed in the same clade when we constructed a phylogenetic tree for a subset of the fungal species recently analyzed by Wang et al. [21]. To our knowledge, from currently available sequence data, this clade is unique to these two fungal species (Figure 7); no similar fungal ABC transporter has been reported.

## Discussion

The ecological niches of saprophytic and pathogenic wood-inhabiting filamentous fungi differ in moisture content, nutrients and defence chemicals. For such fungi to survive in and colonize their substrates and hosts, they need active transport systems that can excrete enzymes that break down complex external substrates and then import nutrients into the cells. As well, they need to modify or remove toxic host defense chemicals that have entered their cells. The wood of trees, logs and lumber has a wide range of moisture contents and a high carbon-to-nitrogen ratio [14]. *O. piceae* grows more efficiently in drier pine wood than in freshly cut logs; *G. clavigera*, which is vectored by MPB, colonizes healthy or stressed living pine trees, which have high moisture and low oxygen contents. Neither organism degrades lignocellulosic wood fibers [1,45]. *O. piceae* has to retrieve nutrients from a nutrient-poor substrate that typically contains very little nitrogen and relatively low levels of host defence chemicals. In contrast, *G. clavigera* has first to cope with high concentrations of defense chemicals produced by its pine host.

Recently, we reported the annotated genome sequence and transcriptomes of the pine pathogen *G. clavigera*[8]. Here, we report the annotated genome and transcriptomes of the saprophyte *O. piceae*, the second pine wood-inhabiting ophiostomatoid fungus for which a complete genome has been sequenced. *O. piceae*’s genome size and the number of predicted genes and proteins were similar to those for *G. clavigera* and other sequenced saprophytic ascomycetes in the class Sordariomycetes (e.g. *N. crassa*, *T. reesei*). *O. piceae*’s predicted secretome is 10% larger than that of the pine-specific pathogen. Given its more diverse range of host trees (e.g. pines, hemlock, spruces), it is likely that the saprophyte requires more extracellular enzymes to degrade the different chemicals encountered in these substrates.

In both genomes we identified genes that are potentially involved in the biosynthesis or processing of SMs. In fungi, SMs are diverse and play a range of roles; some SMs are protective, while others are virulence factors [46]. Both *O. piceae* and *G. clavigera* produce the SM melanin in artificial media and in their natural substrates. Fungal melanin may protect cells in harsh environments (e.g. UV radiation, extreme temperatures and toxic compounds), and may be involved in cellular development, differentiation and pathogenicity [36]. In all conditions tested here, except with terpene treatments, *O. piceae* mycelia and asexual structures (i.e. synemata) were highly melanized. Scytalone dehydratase, which is a marker gene for the DHN pathway [47], was up-regulated in all conditions tested except with terpene treatments, and was most highly expressed in sawdust. Similarly, in *G. clavigera*, which is densely melanized in its pine host, scytalone dehydratase was down-regulated on CM with terpenes, but was up-regulated on other media and when monoterpenes were the only carbon source. In contrast to *O. piceae*, *G. clavigera* does not produce large numbers of asexual spores when it is actively growing on these media. It is likely that melanin protects *O. piceae* from the unfavourable environmental conditions that it encounters in lumber (e.g. dessication, UV), as well as being involved in cellular development. In contrast, for *G. clavigera*, melanin may be more important in protecting the fungus from host defense chemicals.

*O. piceae* and *G. clavigera* can grow on a variety of simple sugars that are present in phloem or in sapwood parenchyma cells [13], and can acquire additional sugars by degrading wood hemicelluloses [13,14,45]. Both fungi grow well with mannose and maltose, and can also use starch, a stored tree nutrient [48]. For *O. piceae*, our data suggest that mannose uptake and the initial steps in its utilization are controlled by at least six genes that include two transporters. That none of the six were up-regulated with maltose suggests that maltose utilization involves an alternate pathway.

*O. piceae* and related species can use triglycerides and fatty acids in artificial media or wood; these lipids can account for up to 3% of the dry weight of sapwood [41]. Triglycerides are hydrolyzed by extracellular lipases into fatty acids and glycerol, which are ultimately processed through ß-oxidation and glycolysis pathways [47]. While lipase and esterase genes were present in the *O. piceae* genome and we noted that a lipase was expressed on sawdust, we were unable to detect up-regulated lipases on triglycerides. It is possible that on triglycerides the lipase was produced very early in growth, as shown by Gao and Breuil [49], who reported an optimum production of the enzyme at day 3, before the pH of the medium decreases due to the accumulation of fatty acids. Here, we collected the mycelium after seven days of growth on a solid media with triglycerides. We identified a glycerol kinase that was up-regulated for triglycerides and sawdust, which suggests that glycerol may be metabolized by the fungus. Further, we noticed that triglycerides induced a genomic cluster that contained a P450 and a reductase (described in Results). Lah *et al.*[35] reported a similar genomic cluster organization and expression pattern in *G. clavigera*, and it is likely that the clusters have similar roles. Lah *et al.* suggested that the cytochrome P450 and the reductase may be specific redox partners and may play a role in the conversion of exogenous phenolics or fatty acids.

*O. piceae* retrieves and metabolizes diverse nutrients that are present in low concentrations in sawdust, particularly nitrogen sources, while removing or modifying diverse toxic compounds like terpenes, and aromatic compounds that include simple phenolics. While the fungus grows more slowly on sawdust, diverse transporters were up-regulated. Some of these are involved in acquiring nutrients like sugars and nitrogen, while others, like ABC or MFS transporters, are known to contribute to drug resistance or chemical modification or detoxification [50].

While small amounts of simple sugars are available in sapwood, *O. piceae* can retrieve additional sugars by degrading pine hemicellulose [13,45]. Fleet et al. [13] reported that mannose was the most depleted sugar in logs and lumber inoculated with *Ophiostoma* species. In our data, the genes up-regulated on sawdust also included glycoside hydrolases (e.g. two xylanases and one pectinase), which are involved in degrading hemicellulose and pectin. As well, the fungus can retrieve quinic acid through a quinate permease, and can utilize this carbon source by processing it through the quinate/shikamate pathway, which was up-regulated on sawdust. Further, in artificial media *O. piceae* can readily use inorganic or organic nitrogen. However, in pine sapwood nitrogen is found mainly as amino acids and proteins, and at very low concentrations (~0.05% of the wood dry weight) [51]. We have shown that *O. piceae* and related species have to produce proteases in order to retrieve organic nitrogen from wood [52]. In the current work, an amino acid permease, and urea and ammonium transporters were up-regulated on sawdust. Urea can be used as a source of nitrogen by many fungi, and it can be efficiently converted into ammonium by a urease enzyme [53]. However, while ammonium is present in trace amounts in pine lumber [53,54], urea has not been reported in wood.

Mono- and diterpenes are well known biocides for microorganisms, including fungi, and for beetle vectors [21,55]. Our data show that on artificial media *O. piceae* tolerates monoterpenes but does not use them as a carbon source. It is not found in living trees, which have the highest terpene concentrations. However, it is able to remain viable for extended periods in the presence of monoterpenes, and likely in the presence of diterpenes, which can account for ~0.4% of pine sapwood dry weight [41]. Here, we show that monoterpenes affected the macroscopic morphology of *O. piceae*’s mycelia, and inhibited its production of synemata and asexual spores. Further, in the saprophyte, monoterpene/diterpene treatments rapidly up-regulated expression of genes involved in oxidative and reductive processes, as well as transmembrane transport, suggesting that the fungus’ primary response involves protecting itself from these chemicals. During these processes, an ABC transporter (OPP_06758), which is homologous to the *G. clavigera* efflux transporter characterized by Wang et al. [21], was highly expressed.

We have shown that this *G. clavigera* ABC-G transporter is expressed in young trees and that the transporter excretes monoterpenes [21]. As we have not yet demonstrated this function for the homologous gene in *O. piceae*, at this time we can only suggest that this unique transporter may play a similar role in the saprophyte by allowing it to survive in toxic mixtures of terpenes. When *O. piceae* is treated with terpenes on rich media, there is an initial growth delay, after which the fungus resumes its growth. In this growth phase, while genes providing most of the primary protective biological functions were active, genes involved in degrading hydrophobic compounds were up-regulated. This suggests that, like *G. clavigera*, *O. piceae* may be able to modify terpenes into less toxic compounds. However, while *G. clavigera* has a gene cluster that specifically responds to terpenes and is potentially involved in metabolizing terpenes [8], in *O. piceae* we found no such gene cluster. Only five of the 30 genes in this *G. clavigera* cluster had homologs in *O. piceae*, and these five genes were dispersed through the *O. piceae* genome. In ongoing work we are characterizing *O. piceae* genes that are involved in modifying terpenes.

## Conclusions

We compared the genomes of *O. piceae* and *G. clavigera*. While we found no large-scale synteny, the ecological niches of both fungi involve growing in pine wood, and both produce similar sets of diverse enzymes. Neither fungus produces a complete battery of cell-wall degrading enzymes, and neither affects the structural properties of wood. We began to clarify differences between the saprophyte and the pathogen, focusing on ABC transporters, CYP450s, genes that produce secondary metabolites like melanin, genes involved in lipid metabolism, and genes that detoxify terpenes and phenolics. *G. clavigera*, but not *O. piceae*, can use monoterpenes as a carbon source. However, both *O. piceae* and *G. clavigera* have a similar ABC-G transporter that, for both fungi, may play an important role in reducing the intracellular concentration of toxic compounds like monoterpenes. Similar specialized transporters may have evolved in other ophiostomatoid fungi that are vectored by insects and inhabit the phloem and sapwood of living or processed conifers.

## Methods

### Strain and growth conditions

The *O. piceae* strains used in this work, either for the genome sequencing or for the mating experiments, had been isolated from freshly sawn timber of *Pinus contorta* at Prince George in British Columbia (Canada) [6]. The strains have been deposited at the University of Alberta Microfungus Collection and Herbarium; ID: UAMH-11346 for the genome sequenced and ID: UMAH-11672 for the mating experiments or for checking the growth characteristics. For growth and maintenance, spores or plugs of fungal mycelium were inoculated and grown at room temperature on plates of MEA (1.6% Oxoid malt extract agar and 1.5% technical agar, pH 5–6). Growth and utilization of terpenes were performed as previously described by Wang et al. [21]. The only exception was for growth on sawdust where spores were inoculated and germinated on 1% MEA (Difco) for 2 days, and then transferred on sawdust plates (15% lodgepole pine sawdust, mixed with 2% granulated agar) overlaid with cellophane for one week. Growth experiments with or without terpenes or with different carbon sources were at least repeated or carried out three times. For RNA-seq analysis, fungal hyphae grown on MEA for three days were transferred to the respective treatment conditions as shown in Table 5.

**Table 5:** Growth conditions for RNA-seq

**Medium**	**Carbon sources or treatment**	**Duration**
CM	No treatment	14 h
CM	No treatment	40 h
CM	200 μl Terpene blend	14 h
CM	200 μl Terpene blend	40 h
YNB	Mannose (1% w/v)	5 days
YNB	*TG: Olive Oil (1% v/v)	5 days
YNB	Oleic acid (0.5% v/v)	5 days
Sawdust	No treatment	1 weeks

* Triglyceride: Olive oil 80% + Fatty acids 20%.

All treatment times were calculated from the initial transfer of actively growing hyphae onto the appropriate medium. CM = 0.17% yeast nitrogen base without amino acids, 1.5% granulated agar, 1% maltose, 0.1% potassium hydrogen phthalate, 0.3% asparagine. YNB = 0.67% yeast nitrogen base without amino acid and 1.5% agar with carbon sources indicated as treatment. The terpene blend was a mixture of monoterpenes and diterpenes as described by Lah et al. [35].

### Genome sequencing

DNA was extracted from fungal hyphae grown on MEA using methods described by Haridas and Gantt [56]. We used two sequencing technologies: Illumina HiSeq 2000, which generated 100 nt reads, and 454 Titanium, which generated reads with a mean length of 318 nt. The libraries for Illumina HiSeq 2000 had two different insert sizes, 200 and 700 nt, while the library for 454 had an insert size of 8000 nt. Illumina HiSeq sequencing was done at the BC Genome Sciences Centre in Vancouver, Canada and 454 sequencing was done at the Plate-forme d’Analyses Génomiques at Laval University in Québec, Canada.

### Genome assembly

Reads generated by the two platforms were used with no further processing for genome assembly using ABySS v1.3.0 [25] with a kmer size of 60. This assembler filters the FASTQ sequences based on quality scores. In order to efficiently use the 454 reads for scaffolding, we used a minimum contig size (1000) and read pairs for building scaffolds (2) (SCAFFOLD_OPTIONS = ‘-s1000 -n2’). The assembly was scrubbed and gaps closed with Anchor (v0.3.0; http://www.bcgsc.ca/platform/bioinfo/software/anchor). When Abyss is unable to find overlaps between contigs where paired end data suggests that the contigs should overlap, it joins the contigs with a single lowercase ‘n’. Such overlaps were resolved using transcriptome assembly (described below) or by finding small overlaps at the ends of the contigs using exonerate v2.2.0 [26]. Genome synteny was assessed by MUMmer [57].

### Transcriptome assembly

RNA-seq was performed on eight RNA samples extracted from the mycelia of *O. piceae* hyphae grown under various conditions as shown in Table 5. For each RNA-seq library, we collected samples from three biological replicates, extracted RNA separately from each replicate, and pooled the samples for paired-end sequencing on an Illumina HiSeq (Canada’s Michael Smith Genome Science Center, Vancouver). Multiplexed sequencing with three libraries per lane was done using the Illumina HiSeq platform to obtain 100 bp paired end reads from 250 bp fragments. Reads were analysed using fastqc (http://www.bioinformatics.babraham.ac.uk/projects/fastqc/) and showed read bias and in the first few bases of the reads and poor quality in the last few. Reads with minimum (Phred) quality scores less than 20 were removed and the first six and last four bases of all reads were trimmed using prinseq [58]. Processed RNA-seq reads were assembled using Trinity [28] using the jaccard_clip option to minimize fusion transcripts. The best protein coding transcripts were identified using the included scripts and aligned back to the assembled genome using exonerate v2.2.0 est2genome [26].

### Genome annotation

We used the Maker annotation pipeline (v2.26) for genome annotation [31]. In addition to the trinity assembled best candidates, we also used two additional sources of evidence in Maker. The first was transcripts predicted by the Core Eukaryotic Genes Mapping Approach [27], (CEGMA) and the second was coding sequences of transcripts assembled by cufflinks [59] from RNA-seq reads mapped to the assembled genome. Within the Maker framework, we trained SNAP v2006-07-28 [60] using the Trinity assembled transcripts, gene models of *Magnaporthe grisea* for Augustus (v2.5.5) [61] and an hmm file for Genemark-ES (v2.3) [62] using an independent run. The UniProtKB/Swiss-Prot (release 2012_01) fasta file was provided as protein homology evidence and pred_flank was set to 50 to minimize fusion transcripts. Predicted genes smaller than 100 amino acids were removed unless they were at least 80 amino acids long and had transcript, protein or CEGMA evidence. Selected gene models were manually curated. Functional identification of predicted genes was done using Blast2go (v2.5.1) [63]. tRNA’s were identified using tRNAscan-SE (v1.3.1) [64]. Relative synonymous codon usage (RSCU) was calculated using a local installation of the graphical codon usage analyser [65]. Secretome predictions were made with TargetP [66] and Phobius [67]. A protein was considered to be secreted if either TargetP or Phobius suggested that it was secreted and this result was not in conflict with the other. Identification of secondary metabolism genes and clusters was done using the Secondary Metabolite Unique Regions Finder (SMURF) [34].

### RNA-Seq analysis

Quality trimmed RNA-seq reads were aligned to the *O. piceae* genome using Bowtie (v0.12.7), Tophat (v2.0.4), Cufflinks (v2.0.2) as described by Trapnell et al. [59]. Because mapping the RNA-seq reads to the genome without providing fixed gene models resulted in an unacceptable number of predicted fusion transcripts, reads were mapped using the curated gene models predicted by the Maker pipeline.

### Data availability

The sequences reported in this paper are being deposited in NCBI gene bank as assembly and annotations, Project NO. PRJNA182071.

## Abbreviations

Mbp: Mega base pair; MPB: Mountain pine beetle; nt: Nucleotide; bp: Base pair; UTR: Untranslated region; CDS: Coding sequence; SM: Secondary metabolite; NRPS: Nonribosomal peptide synthase; PKS: Polyketide synthase; DHN: 1,8-dihydroxynaphthalene; FPKM: Fragments per kilobase of exon per million fragments mapped; MFS: Major facilitator superfamily; YNB: Yeast nitrogen base; MT: Monoterpene; MEA: Malt extract agar; CM: Complete medium; UV: Ultra violet; CEGMA: Core Eukaryotic Gene Mapping Approach; RSCU: Relative synonymous codon usage; SMURF: Secondary Metabolite Unique Regions Finder.

## Competing interests

The authors declare that they have no competing interests.

## Authors’ contributions

SJ and RD performed the initial genome assembly, and SH the final assembly. SH annotated gene models, analyzed transcriptomes, and drafted the results for the manuscript. YW participated in and finalized the transcriptome analyses. LL prepared DNA, mRNA, performed growth experiments and participated in the analyses. SMA did the phylogenetic trees and micrographs. IB supervised the sequencing of the genome and transcriptomes. GR, JB and CB wrote and completed the manuscript. CB and JB conceived and directed the project. All co-authors critically reviewed, edited and approved the manuscript.

## Supplementary Material

Additional file 1 **Comparison of the genomes of *O. piceae *and *G. clavigera, *using MUMmer**[57]**.** A dot plot genomic comparison showed no large scale synteny between the assembled genomes of *O. piceae* and *G. clavigera*. The genome of *O. piceae* is represented on the X-axis and that of *G. clavigera* on the Y-axis. Click here for file

Additional file 2 **GO term enrichment in a set of 3,469 genes of *O. piceae *which were not reciprocal best blast hits in the *G. clavigera *genome.** #Test represents the number of genes annotated with the respective GO term in the set of 3,469 genes and #Ref represents the number of genes with the GO classification in the entire *O. piceae* genome. P-value is calculated for Fisher’s exact test and FDR is the false discovery rate. Click here for file

Additional file 3 **The eleven secondary metabolism clusters in *O. piceae *predicted by SMURF **[34]**.** The backbone gene in each cluster is highlighted in yellow. Click here for file

Additional file 4 **Expression patterns of the 677 significantly differentially regulated genes.** Expression values measured as FPKM (fragments per kilobase of exon per million fragments mapped). Genes that showed a 10 times or greater up-regulation in one condition or a related set of conditions are colour coded as described. Click here for file

Additional file 5 **Alternative splicing.** To assess how important alternative splicing and transcripts were, we used Tophat and Cufflinks to map the RNA-seq reads to the genome assembly using the techniques described by Trapnell et al. The results suggested that approximately 150 alternative transcripts were expressed; however, all of these appeared to be false positives. The dominant cause of these false positive predictions was that closely spaced genes with overlapping UTRs were misassembled as single contigs, and differential regulation of such genes under different growth conditions appeared as alterative isoforms. In other cases, mapping errors produced false gene calls and alternative isoforms. These results indicated that alter415native splicing is not important under the growth conditions used for this study. Click here for file

Additional file 6 **Growth of *O.piceae *and*G. clavigera *on mannose, oleic acid and quinic acid.** Fungal plugs were inoculated onto YNB plates (pH ~ 7, adjusted by KH_2_PO_3_-K_2_HPO_3_ buffer) containing a single carbon source; the plates with the fungus were incubated for 2 weeks. The growth of *O. piceae* is slower than *G. clavigera*, as shown with mannose. Control: YNB with no carbon. Click here for file

Additional file 7 **Growth of *O. piceae *on MEA with or without the addition of monoterpenes for a week. A**) Growth on MEA alone (arrow a: synemata and spores, arrow b: mycelium), **B**) Growth on MEA with monoterpenes, the mycelium was more aerial and fluffy (arrow c) while the production of asexual structures was highly inhibited. Click here for file
