# Supplementary figures and images for: The genome and transcriptome of the pine saprophyte Ophiostoma piceae, and a comparison with the bark beetle-associated pine pathogen Grosmannia clavigera
Source: BMC Genomics. 2013 Jun 2;14:373. doi: 10.1186/1471-2164-14-373 (PMC3680317; doi:10.1186/1471-2164-14-373)

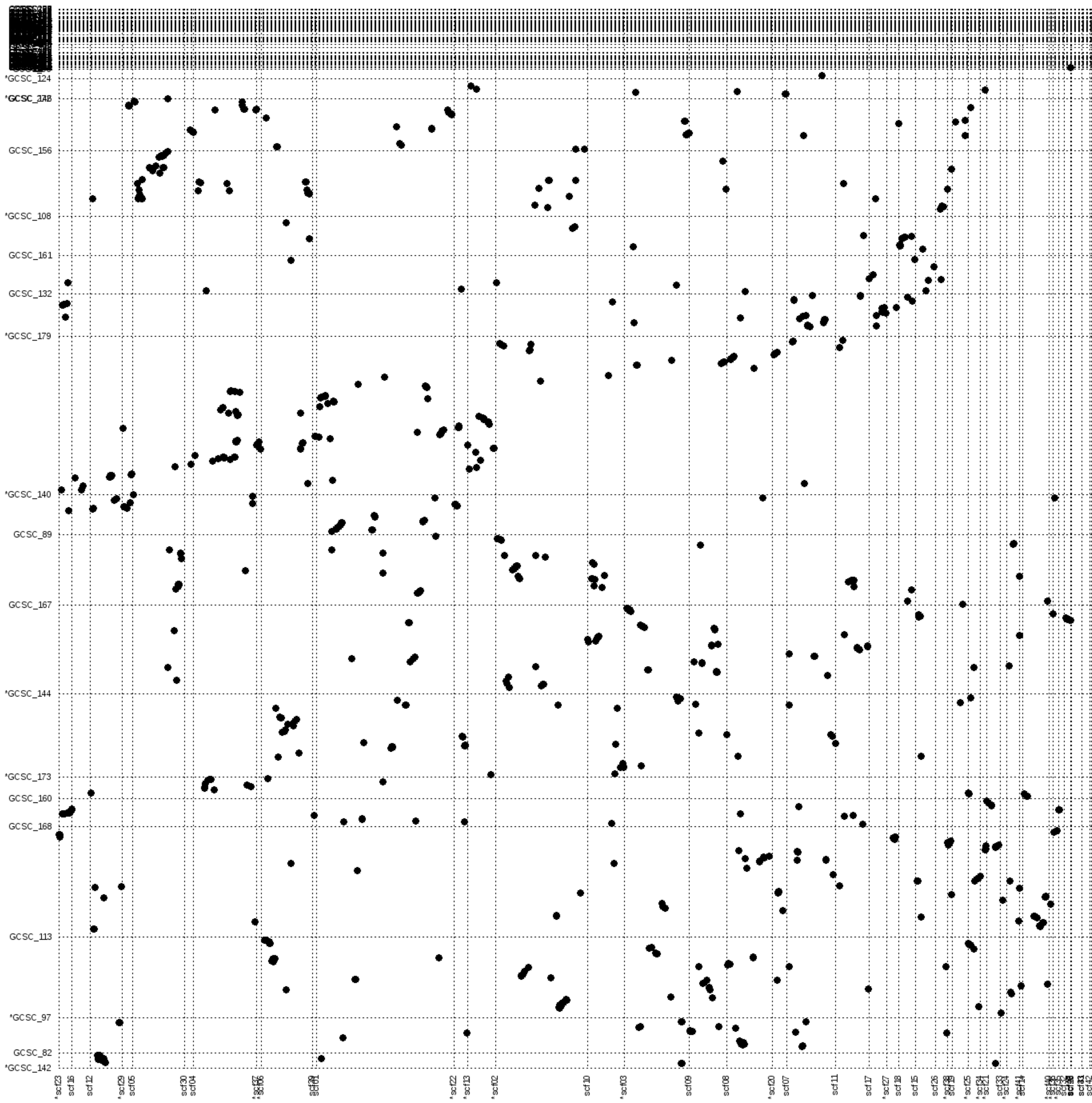

Supplement: Additional file 1 — Comparison of the genomes of O. piceae and G. clavigera, using MUMmer[57]. A dot plot genomic comparison showed no large scale synteny between the assembled genomes of O. piceae and G. clavigera. The genome of O. piceae is represented on the X-axis and that of G. clavigera on the Y-axis. [file 1471-2164-14-373-S1.pdf]

Control

1% Mannose

0.5% Oleic Acid

0.5% Quinic Acid

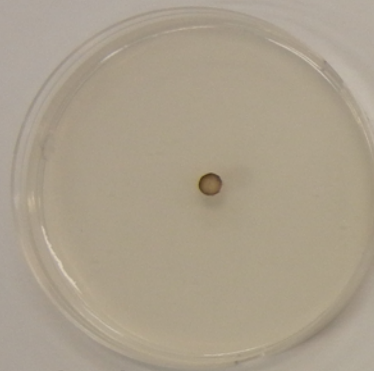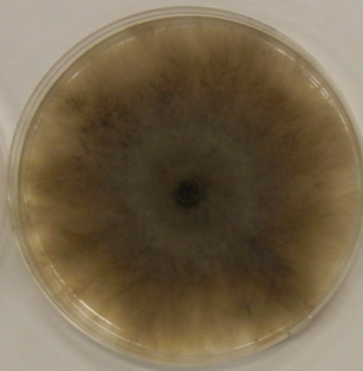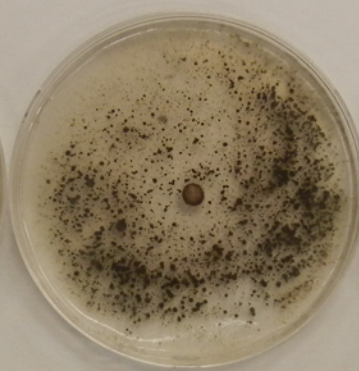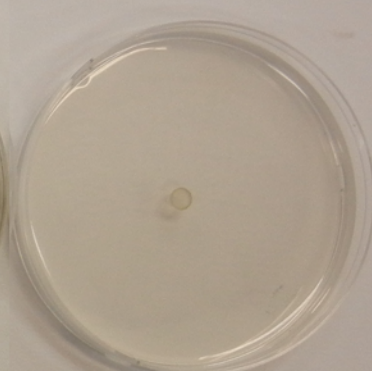

*G. clavigera*

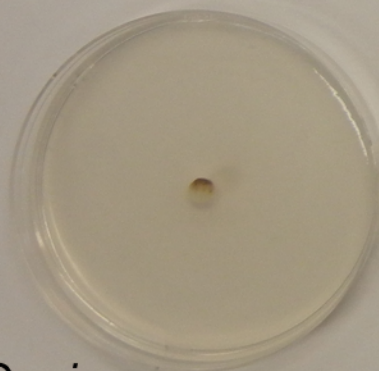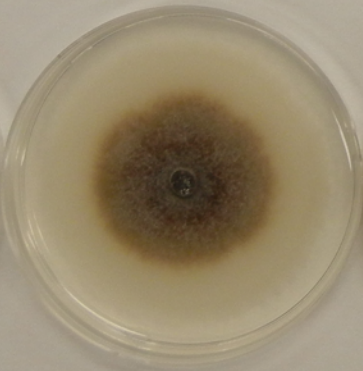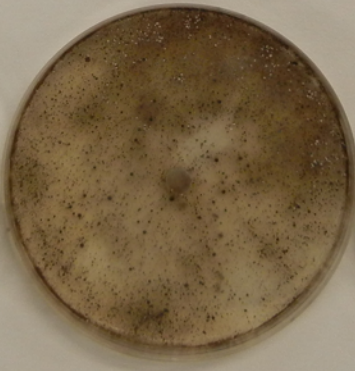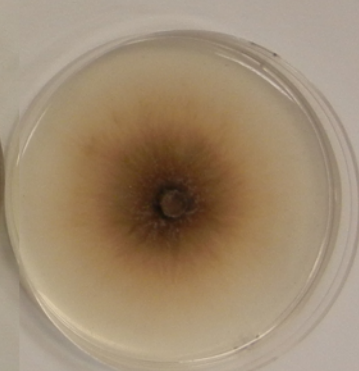

*O. piceae*

Supplement: Additional file 6 — Growth of O.piceae andG. clavigera on mannose, oleic acid and quinic acid. Fungal plugs were inoculated onto YNB plates (pH ~ 7, adjusted by KH2PO3-K2HPO3 buffer) containing a single carbon source; the plates with the fungus were incubated for 2 weeks. The growth of O. piceae is slower than G. clavigera, as shown with mannose. Control: YNB with no carbon. [file 1471-2164-14-373-S6.pdf]

**A**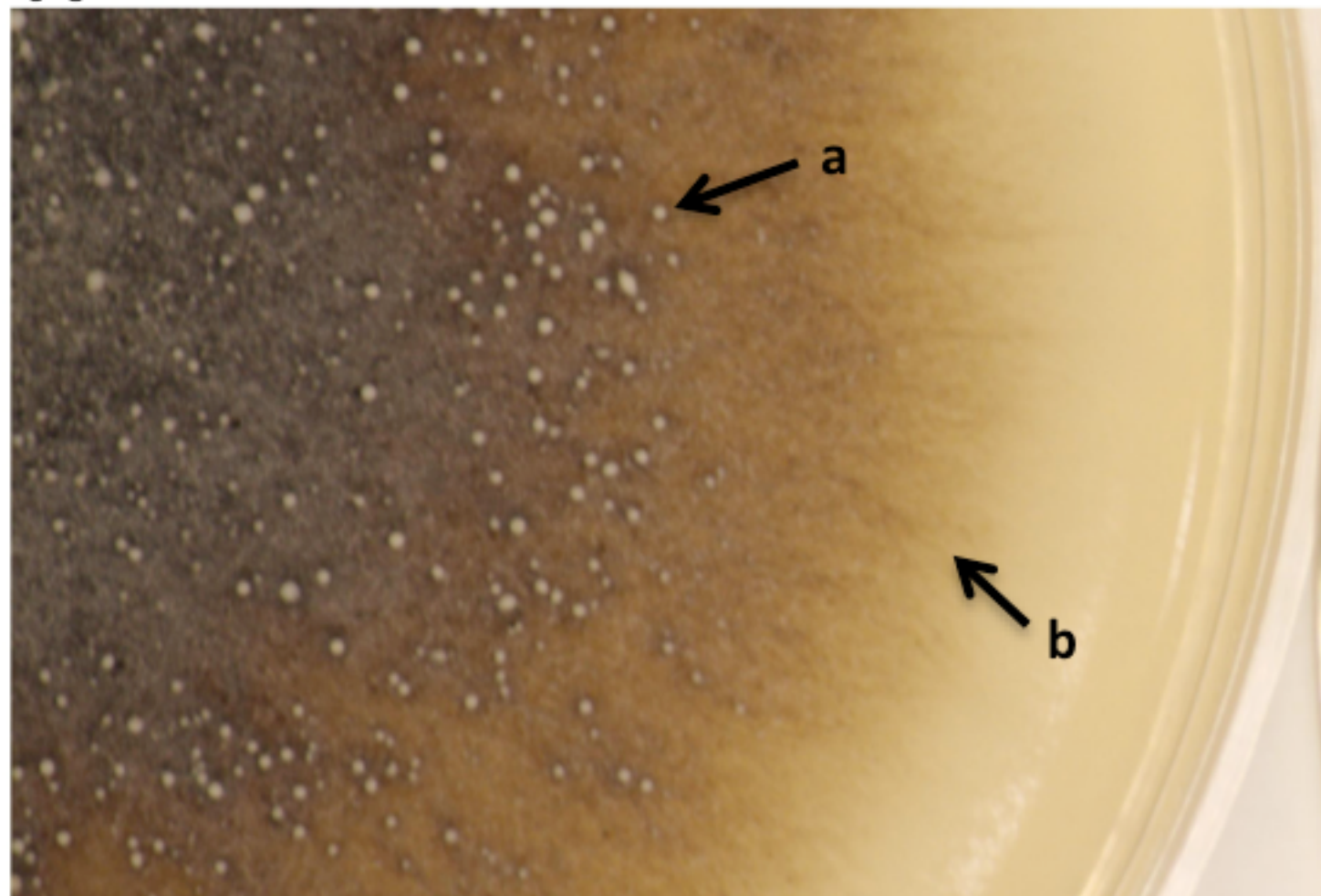**B**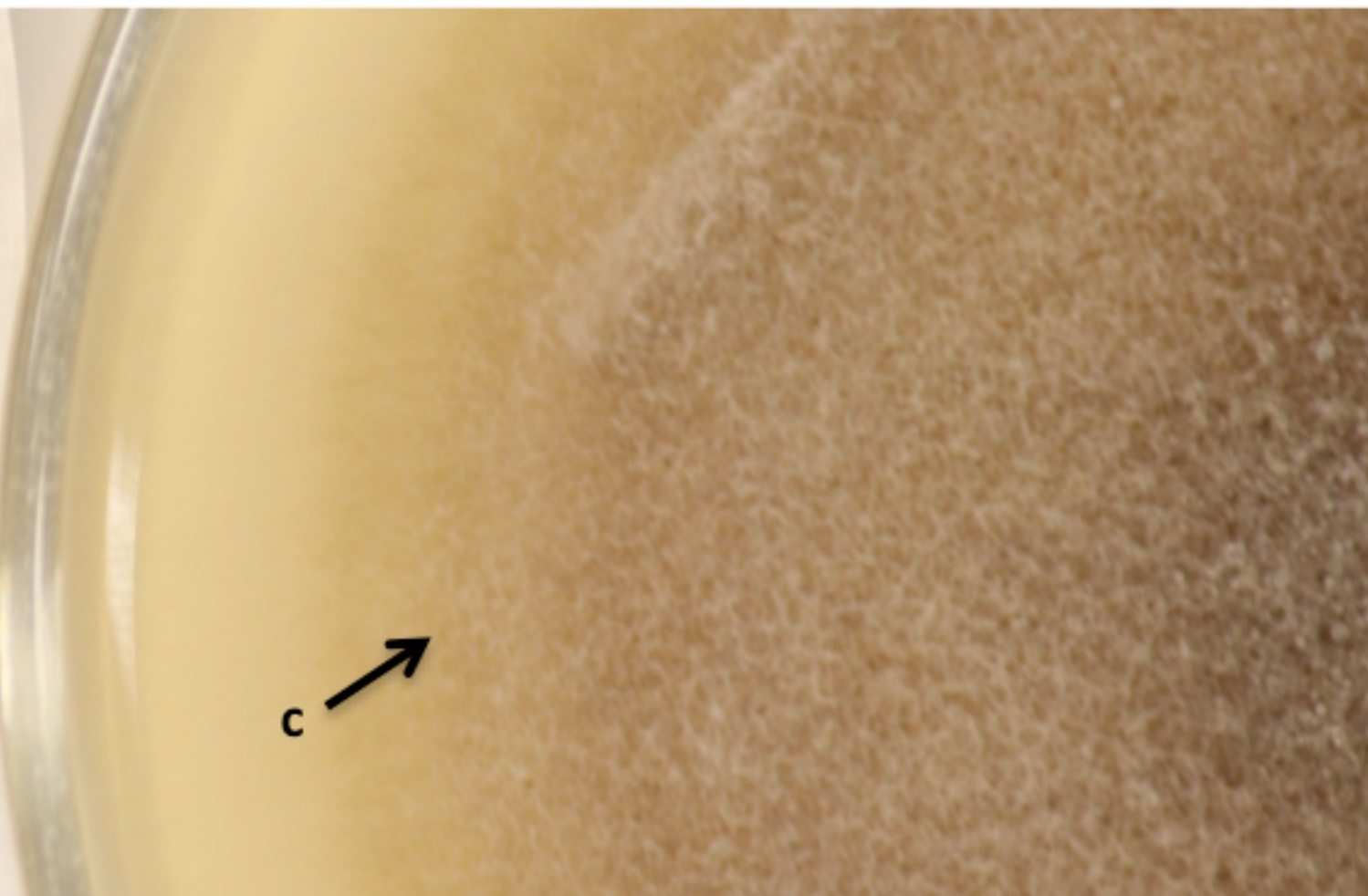

Supplement: Additional file 7 — Growth of O. piceae on MEA with or without the addition of monoterpenes for a week. A) Growth on MEA alone (arrow a: synemata and spores, arrow b: mycelium), B) Growth on MEA with monoterpenes, the mycelium was more aerial and fluffy (arrow c) while the production of asexual structures was highly inhibited. [file 1471-2164-14-373-S7.pdf]
