# Supplementary material for: The genome and transcriptome of the pine saprophyte Ophiostoma piceae, and a comparison with the bark beetle-associated pine pathogen Grosmannia clavigera
Source: BMC Genomics. 2013 Jun 2;14:373. doi: 10.1186/1471-2164-14-373 (PMC3680317; doi:10.1186/1471-2164-14-373)
Supplement: Additional file 5 — Alternative splicing. To assess how important alternative splicing and transcripts were, we used Tophat and Cufflinks to map the RNA-seq reads to the genome assembly using the techniques described by Trapnell et al. The results suggested that approximately 150 alternative transcripts were expressed; however, all of these appeared to be false positives. The dominant cause of these false positive predictions was that closely spaced genes with overlapping UTRs were misassembled as single contigs, and differential regulation of such genes under different growth conditions appeared as alterative isoforms. In other cases, mapping errors produced false gene calls and alternative isoforms. These results indicated that alter415native splicing is not important under the growth conditions used for this study. [file 1471-2164-14-373-S5.doc]

**Additional file 3.** Alternative splicing

To assess how important alternative splicing and transcripts were, we used Tophat and Cufflinks to map the RNA-seq reads to the genome assembly using the techniques described by Trapnell et al. The results suggested that approximately 150 alternative transcripts were expressed; however, all of these appeared to be false positives. The dominant cause of these false positive predictions was that closely spaced genes with overlapping UTRs were misassembled as single contigs, and differential regulation of such genes under different growth conditions appeared as alterative isoforms. In other cases, mapping errors produced false gene calls and alternative isoforms. These results indicated that alternative splicing is not important under the growth conditions used for this study.
